# Application of comfort nursing in mother-infant room exerts beneficial effects

**DOI:** 10.4314/ahs.v23i2.83

**Published:** 2023-06

**Authors:** Yan Wang, Jingjing Li

**Affiliations:** 1 The First People's Hospital of Lianyungang, Lianyungang 222001, Jiangsu Province, China; 2 Lianyungang Hospital Affiliated to Xuzhou Medical University, Lianyungang 222002, Jiangsu Province, China

**Keywords:** Mother-infant co-living, comfort care, application, growth, development

## Abstract

**Objective:**

To analyse the clinical application of comfort care in mother-infant room.

**Methods:**

A total of 150 cases of maternal and infant delivery in the same room were selected for the study, randomly divided into two groups, the experimental group and the control group were 75 cases. The control group took conventional nursing intervention measures, and the experimental group implemented the comfortable nursing intervention mode. Under the guidance of comfort concept, nursing management was carried out from environment, physiology, psychology and other aspects. The postpartum negative emotions, comfortable conditions, the growth and development of newborn babies and the satisfaction of nursing work were compared.

**Results:**

After intervention, experimental group had lower scores of HAMD and HAMA as well as higher GCQ scores than those of control group (P < 0.05). The body length, body weight and head circumference of neonates, as well as nursing technology, professional service and environmental satisfaction in experimental group were higher than those in control group (P < 0.05).

**Conclusion:**

Applying the comfort nursing mode in mother-infant room can help improve the maternal physical and mental states, promote the growth and development of newborn, and markedly improve the quality and efficiency of nursing work.

## Introduction

With the continuous development of medical and health services and enhancement of health awareness, the requirements for obstetrical maternal and infant care have increased. Mother-infant co-living is an important reform of the obstetric system and an effective measure to promote the success of breastfeeding 1-3. After delivery, postpartum fatigue is a risk factor for mothers to lack milk and reduce the rate of breastfeeding. The main causes of postpartum fatigue include postpartum mental stress, poor sleep-in hospital and cesarean section. If nursing intervention is not timely and effective, the physical and mental state of the puerpera will be seriously affected 4,5. Meanwhile, the fetus undergoes great changes after being separated from the mother due to the obvious difference between internal and external environment, so the nursing mode has an important impact on the growth and development of the newborn. On the other hand, lactation may be affected by the lack of professional knowledge of neonatal nursing and daily nursing skills of puerperae and their family members, and by the negative emotions and psychological pressure of puerperae. Hence, teaching the knowledge of neonatal nursing, improving the breastfeeding quality and enhancing nursing satisfaction play key roles in obstetric nursing.

Thereby motivated, the aim of this study was to clarify the effect of comfort nursing on mothers and infants.

## Materials and methods

### Subjects

From December 2019 to December 2020, 150 mother-infant cases who delivered in our hospital were selected in this prospective randomized controlled study. All of them were primiparas with single fetus, and they were divided into two groups by random number method, the experimental group and the control group were both 75 cases. There was no significant difference in the baseline data between the two groups (P>0.05) (Table 1).

**Table 1:** General data of parturient and newborn

Baseline data		Experimental group (n=75)	Control group (n=75)	*x^2^/t*	*P*
Maternal age (year)		26.75±2.16	26.81±2.14	0.171	0.865
Delivery way	Cesarean delivery.	49 (65.33)	52 (69.33)	0.273	0.601
	Natural birth.	26 (34.67)	23 (30.67)		
Degree level	Junior high school and below.	9 (12.00)	7 (9.33)	0.280	0.869
	High school, technical secondary school.	29 (38.67)	30 (40.00)		
	College degree or above.	37 (49.33)	38 (50.67)		
Gestational week of delivery (week)		39.12±0.75	38.97±0.82	1.169	0.244
Newborn sex	Male	46 (61.33)	44 (58.67)	0.111	0.739
	female	29 (38.67)	31 (41.33)		
Birth weight (kg)		3.26±0.75	3.19±0.82	0.546	0.586
Apgar score (points)		7.46±1.45	7.52±1.61	0.240	0.811

**Inclusion criteria:** (1) informed and agreed to the study; (2) the mother and child are in the same room, willing to breastfeed, and there is no taboo against breastfeeding; (3) No serious postpartum complications, good language and communication skills.

**Exclusion criteria:** (1) severe disturbance of complicated vital organs; (2) mental or language disorders; (3) disability or physical or motor dysfunction; (4) Unable to cooperate or quit the study halfway.

### Methods

In the control group, conventional nursing intervention measures were taken. In addition to conventional postpartum nursing measures, U-shaped pillows and footstool were prepared for breastfeeding in the room with mother and infant to create a quiet and comfortable room environment for pregnant women. Optimum indoor temperature and humidity were maintained. The window was opened twice a day for ventilation. The shift of nursing staff was adjusted, APN shift was adopted, and the night shift handover time was 10:00 pm, which did not affect patients' sleep at night and was in line with patients' working and rest habits. Daytime treatment and care should be as focused as possible so as not to disturb and affect other mothers. The neonatologist makes daily ward rounds to assess the condition of the newborn and inform the mother and her family.

The experimental group implemented the comfort nursing intervention mode, guided by the comfort concept, nursing management was carried out from the environment, physiology, psychology and other aspects. Skin-to-skin contact between mothers and infants was performed. Environment: The layout of mother and child room was decorated to conform to their physiology and psychology. The head and tail of the bed can be lifted up and down, equipped with safety gear and baby bed, which is conducive to rest and safety. Some beds and clothes were warm and soft pink and had spare parts; The door of the sickroom is provided with a door stopper and a spring, which eliminates the sound of closing and opening the door; Plastic carpet is laid on the ground, which can prevent skidding and reduce walking sound. There are handrails on both sides of the corridor, and the walls are full of children's paintings for mothers and babies to enjoy. Each ward is equipped with a radio, which can play music, provide a comfortable rest environment, change and clean clothes every day, and bathe the baby every day to meet the requirements of comfort care.

**Physiology:** According to the maternal eating habits and postpartum physical conditions, nutritionists develop postpartum two-point nutrition meals, three meals a day, to provide scientific nutrition meals for the puerpera, to help the puerpera recover strength. Parturients were correctly guided to wash hair, face, brush teeth with warm water and other appropriate activities. According to the needs of pregnant women, their bed sheets and sick clothes were changed at any time to keep them clean and comfortable. Nursing staff should urge the maternal in postpartum 30 minutes in a timely manner to urinate, so as to avoid bladder swelling affecting uterine contraction. When necessary, abdominal massage can be used, hot compress, listen to the sound of water and other methods to promote urination. The rate of breastfeeding was improved to prevent breast pain. Nursing staff carry out three shifts of bed handover every day. The handover focuses on the handover of breastfeeding posture, newborn situation, breast situation, self-feeling during breastfeeding, etc., and timely gives correct breastfeeding guidance for problems.

**Psychology:** The psychological state of the puerpera was objectively and correctly evaluated, the puerpera's questions were answered, the personal habits were respected, scientific puerpera life style was provided, and the husbands and family members were encouraged to actively participate in neonatal care activities. Curtains were closed to protect the maternity's privacy during treatment and care. The newborn's bathing, swimming, touching and other care is carried out in the maternal visible state to reduce unnecessary worry and anxiety 6.

### Evaluation of Efficacy

The Hamilton depression scale was used before and after nursing intervention (Hamilton Depression Scale, HAMD). The higher the score is, the more serious the maternal depression is; the score below 7 is normal; and the score over 24 is severe depression.

The Hamilton Anxiety Scale (Hamilton Anxiety Scale, HAMA) was used to evaluate maternal anxiety. The scale has 14 items, using a 5-level scoring method, 0 is asymptomatic, the higher the score means the maternal anxiety is more serious.

General Comfort Questionnaire (GCQ) was used before and after nursing intervention to estimate the comfort level of puerpera. The scale has four dimensions, including physiological, psychological, socio-cultural and environmental, and spiritual, with a total of 28 items. Take 1 to 4 when scoring Likert scale, 1 means strongly disagree, and 4 means strongly agree. 1 means I strongly agree with you. 4 means I strongly disagree. The higher the score, the higher the comfort level.

The growth and development of the two groups of neonates after intervention were recorded. The main indicators were elongation, weight and head circumference. To investigate the satisfaction degree of parturient women to nursing work, mainly from the nursing technology, service and environment several aspects of evaluation 7.

### Statistical analysis

SPSS19.0 software was used for statistical analysis. Numerical and measurement data were subjected to the χ^2^ and t tests, respectively. P < 0.05 was considered statistically significant.

## Results

### Maternal depression and anxiety before and after intervention

There was no significant difference in the baseline data between the two groups (P>0.05) (Table 1). Before intervention, there was no significant difference in depression and anxiety between the two groups (P > 0.05). After intervention, the HAMD and HAMA scores of the experimental group were lower than those of the control group (P < 0.05) (Table 2).

**Table 2:** Depression and anxiety before and after intervention ( ± s, point)

Group	Number of cases	HAMD		HAMA	

		Before intervention	After intervention	Before intervention	After intervention
Experimental group	75	18.26±2.37	6.55±0.71	17.64±2.16	7.85±0.92
Control group	75	18.19±2.42	7.01±1.02	17.58V2.09	8.52±1.98
*t*		0.179	3.205	0.173	2.737
*P*		0.858	0.002	0.863	0.007

### Maternal comfort before and after intervention

Before intervention, there was no significant difference in postpartum comfort status between the two groups (P > 0.05). After intervention, the GCQ scores of the experimental group were higher than those of the control group (P < 0.05) (Table 3).

**Table 3:** GCQ scores before and after intervention ( ± s, point)

Comfort rating		Experimental group (n=75)	Control group (n=75)	*t*	*P*
Physiological	Before intervention	1.81±0.22	1.79±0.24	0.532	0.596
	After intervention	2.91±0.19	2.76±0.45	2.659	0.009
Psychological	Before intervention	2.03±0.36	2.06±0.34	0.525	0.601
	After intervention	3.86±0.41	3.62±0.51	3.176	0.002
Social culture and environment	Before intervention	2.17±0.43	2.16±0.41	0.146	0.884
	After intervention	3.65±0.46	3.39±0.59	3.042	0.003
Spirit	Before intervention	1.87±0.39	1.85±0.38	0.318	0.751
	After intervention	3.02±0.43	2.76±0.54	3.262	0.001

### Growth and development of neonates after intervention

The body length, body weight and head circumference of neonates in the experimental group were all higher than those in the control group (P < 0.05) (Table 4).

**Table 4:** Growth and development indices of neonates after intervention ( ± s)

Group	Number of cases	Body length (cm)	Weight (kg)	Head circumference (cm)
Experimental group	75	52.46±3.15	4.89±0.64	35.89±1.23
Control group	75	50.89±2.34	4.53±0.75	34.94±2.11
*t*		3.465	3.162	3.369
*P*		0.001	0.002	0.001

### Nursing service satisfaction

The nursing technology, professional service and environmental satisfaction of the experimental group were higher than those of the control group (P < 0.05) (Table 5).

**Table 5:** Satisfaction degrees with nursing service [n (%)]

Group	n	Technology	Professional service	Environment
Experimental group	75	74 (98.67)	73 (97.33)	74 (98.67)
Control group	75	68 (90.67)	66 (88.00)	65 (86.67)
*x^2^*		4.754	4.807	7.946
*P*		0.029	0.028	0.005

## Discussion

The separation of the mother and infant in the same room means that the mother and infant stay together for 24 hours, which can strengthen the emotional communication between the mother and infant and lay a good foundation for subsequent feeding 6-8. Nursing with mother and infant in the same room enables mothers and their family members to participate in the daily care. In the process, they understand the physiological characteristics of the newborn and related health knowledge 9,10.

Comfort care is the “people-oriented” nursing concept, to provide a full range of services, so that patients to achieve the maximum comfort. Comfort care for mothers and infants in the same room takes maternal and newborn as the core, pays attention to the overall physical and mental condition of maternal, and tries its best to satisfy the maternal 11,12. The results of this study show that the application of comfort nursing measures in the room with mother and baby can significantly improve the maternal mood, comfort and growth and development of newborn.

Breast milk contains various substances required for infant growth and development, and considerable growth hormone and immune antibodies, so breastfeeding is currently recognized as the most ideal way of feeding newborns. During the feeding of newborns, mothers should be encouraged to keep breastfeeding. However, they may experience insufficient lactation or breast discomfort, which affects the quality of feeding. Nursing for mothers and infants in the same room can elevate the success rate of breastfeeding, and facilitate the growth of newborns 13-16. Immediately after birth, the newborn is in contact with the mother's skin, which stabilizes the newborn's body temperature, attenuates external cold stimulation, and reduces the number of crying accordingly 17,18. Sucking breast milk as soon as possible after birth can promote the secretion of prolactin and oxytocin in pregnant women, being span style=“font-family: ‘Times New Roman’; -aw-import: spaces”> beneficial to further breastfeeding 19,20. According to the theory of planned behavior, behavior intention can directly affect individual behavior, and the individual's perceived ability to control behavior also directly and indirectly affects the final behavior 21. Based on this, during the implementation of nursing intervention plan, the mother's attitude towards breastfeeding should be well educated. Through individualized guidance, the mother can learn the correct feeding posture and knowledge. Meanwhile, family members are encouraged to participate in this process and give sufficient support.

The application of comfortable nursing in residential nursing can enable puerpera to enjoy the joy of being a new mother in a comfortable and warm environment, guide puerpera to get familiar with relevant nursing knowledge, improve the success rate of breastfeeding as soon as possible, and promote the healthy growth of newborn 22,23. In the nursing process, the nursing staff should not only play a leading role, guide the maternal into the role as soon as possible through practical operation, but also do a good job in cooperation. Through the relevant health guidance, let the puerpera become the main body, undertake the relevant nursing work. It is the responsibility of nursing staff to meet the comfort needs of patients. Comfort nursing can ensure the true recovery and comfort of puerpera and newborns, timely adapt to the discomfort caused by role changes, make mothers and babies safer, healthier and more comfortable, so as to truly realize the nursing tenet of “patient-cantered”.
